# The impact of Ramadan during COVID-19 confinement on weight, dietary, and lifestyle habits in the Kingdom of Saudi Arabia: a cross-sectional study

**DOI:** 10.1186/s12889-022-13953-9

**Published:** 2022-08-30

**Authors:** Mai A. Khatib

**Affiliations:** Dietitian and Assistant professor at the Clinical Nutrition Department, Faculty of Applied Medical Sciences, King Abdulaziz University, P.O. Box 80215, Jeddah, 21589 Saudi Arabia

**Keywords:** Weight, Ramadan, Covid-19, Confinement, Saudi Arabia

## Abstract

Severe procedures were undertaken globally because of the COVID-19 pandemic to overcome the spread of the disease and to prevent catastrophic results affecting the health care system including social distancing, lockdowns, and quarantines. Despite the widely known health benefits of Ramadan fasting, there was a general concern regarding the lifestyle of people during Ramadan 2020 that accompanied the period of COVID-19 pandemic and the home confinement applied. The main objective for the current cross-sectional investigation was to investigate the influence of Covid-19 lockdown during Ramadan fasting on weight change on 481 participants in Saudi Arabia. Identifying the contributing risk factors to weight gain were also addressed. Around 42% of the participants had gained weight and around 38% of the participants had lost weight. Physical activity level was shown to be considered as a protective factor against weight gain (OR = 1.03 with *P* = 0.008), while increasing the number of meals and not adapting healthy cooking methods can both be considered as contributing factors to weight gain (OR = 1.03 with *P* = 0.009, and OR = 1.03 with *P* = 0.004, respectively). Assessing these changes during Ramadan of COVID-19 quarantine provided valuable perspective on the health and wellbeing of Saudi Arabia citizens. These findings should be considered in future studies to explore the persistence of Covid-19 related weight status and habit change.

**Supplementary Information:**

The online version contains supplementary material available at 10.1186/s12889-022-13953-9.

## Introduction

Severe procedures were undertaken globally because of the COVID-19 pandemic to overcome the spread of the disease to prevent catastrophic results affecting the health care system including social distancing, lockdowns, and quarantines. The health status of a particular population is a global public health concern, especially with these restrictive policies that limited access to health care systems, transportation to worksites, changed physical activity level, and changed the quality of dietary intake [1, 2]. Yet, the influence of these confining procedures on weight is not fully elucidated, with various findings were shown globally [3–6]. Examining obesity risk factors during this critical period of pandemic-related lockdown is important, especially with the lack of weight management treatment worldwide [7]. Identifying obesity contributors on the population level is essential from a policy perspective, to help determine effective strategies targeted against weight gain when such restrictive procedures are intended to be applied again in the future.

A series of curtailments on individual movements have been applied by the Saudi Arabian government. During the partial lockdown, people were permitted to leave their homes only from 06:00 to 19:00, whereas permission to leave homes for reported causes such as grocery shopping in the neighbourhood was only from 06:00 to 15:00 during total lockdown. The home quarantine period started on the 23^rd^ of March and ended on the 21^st^ of June 2020 [8], during which the holy month of Ramadan is. Ramadan fasting is considered in the scientific literature as a type of intermittent fasting, which is an obligatory spiritual practice for Muslims that involves abstaining from eating, drinking, intercourse, and other activities from sunrise to sunset [9, 10].

Lockdown can alter nutritional habits, and cause lifestyle disturbances such as increased daily sitting time [1, 2, 11, 12]. On the other hand, Ramadan month is considered as a good chance by Muslims to have lifelong influences by making meaningful modifications to their lifestyle in general that empower them to live a happier and healthier life with their beloved ones [13]. In addition, it improves an individual’s self-discipline and self-control [13]. Despite the widely known health benefits of Ramadan fasting, there was a general concern regarding the lifestyle of people during Ramadan 2020 that accompanied the period of the home confinement applied because of the COVID-19 pandemic. The present study highlighted the cross-sectional effect of the lockdown and home confinement applied during Ramadan 2020 on weight status and lifestyle changes among people living in the Kingdom of Saudi Arabia (KSA). Therefore, the main aim of the current research was to investigate the influence of Covid-19 confinement during Ramadan fasting on weight change. Secondary objectives were to identify the most preventive and contributing risk factors to weight loss and/or weight gain, if any.

## Methods

### Design of the study and study participants

The present cross-sectional study took place between May 17 and May 23, 2020, which was the last week of Ramadan holy month (April 23-May 23, 2020). Within this period of time in KSA, number of confirmed cases of Covid-19 has more than doubled to 98,869 from 41,014 (John Hopkins Corona Center; https://coronavirus.jhu.edu/) [14]. The restrictions imposed in Saudi Arabia with respect to timeline of events are shown in Table 1. Eligibility to voluntarily participate in the study was considered if participants were adults (>18 years old) of Saudi and non-Saudi nationalities who resided in the country and were with access to internet. Participants’ email addresses were collected to avoid duplication of data entry. Additionally, volunteered participation was accepted after obtaining written informed consent from all respondents. The Ethics Committee for Post Graduate Studies and Scientific Research at the College of Applied Medical Sciences, King Abdulaziz University, KSA, has approved the research design and protocol (reference number FAMS-EC2020-004). All methods were applied according to relevant regulations and guidelines.

**Table 1:** Restrictions applied in Saudi Arabia with respect to timeline of events

Event	Dates	Curfew hours	Exception
Suspension of non-essential work	Mar 15	None	None
Nationwide curfew	Mar 23–Apr 5	6AM–7PM	Makkah/Madinah
Enhanced curfew	Apr 6–25	6AM–3PM	None
Ramadan^a^	Apr 26–May 22	9AM–5PM	Makkah
Eid Al Fitr	May 23–27	24 h	None
Phase 1 partial easing	May 28–30	6AM–3PM	Makkah
Phase 2 partial easing	May 31–Jun 20	6AM–6PM	Makkah

^a^ Denotes events happened throughout data collection timeline (May 17–May 23, 2020). Table adapted from Alfawaz and their colleagues [15].

### Questionnaire

Collection of data was done via an online electronic survey and all of the included participants completed filling the electronic survey within Ramadan (100%). The questionnaire included 26 questions and started with a cover letter in Arabic language that explained the purpose of the study, information to reach out for the principal investigator, and the consent form. Questions were included based on what was mentioned in the literature and according to study objectives. The electronic survey consisted of demographic and social information, and multiple-choice questions to determine the dietary habits changes related to COVID-19, and lifestyle in general, including physical activity, screen time, smoking, and sleeping habits among participants. Moreover, changes in weight in kg since COVID-19 quarantine started and on-time of questionnaire collection was also requested to be filled, subjectively. Questions were summarised in Table 2 and were attached to supplementary material. Experts of medical background who were resided in the country critically appraised the electronic survey and various revisions were done to add to the scientific value of the data to be collected along with increasing the validity and reliability of the survey questions. Both the language and the cultural correspondence were reviewed for all questions and answers. Whenever answers were not applicable or not mentioned, notes were taken and edited form was revised in the original survey. The validity and reliability of the electronic questionnaire was confirmed with the aid of a pilot study (*n* = 5), the results of which were not included in the larger scale study. Afterwards, the electronic survey was distributed to several social media portals to reach different and the furthest areas of KSA.

**Table 2:** Characteristics of the studied population (*n* = 481)

Parameters			Frequency (%)
Gender		Female	297 (61.7)
		Male	184 (38.2)
Age		18–25 years	53 (11.0)
		26–35 years	153 (31.8)
		36–45 years	90 (18.7)
		> 45 years	205 (42.6
Body Mass Index (at the time of data collection)		Underweight	12 (2.49)
		Normal weight	162 (33.67)
		Overweight	159 (33.05)
		Obese	148 (30.76)
Sociodemographic parameters			
Education level		High school	62 (12.4)
		Diploma	6 (1.2)
		Bachelor’s degree	323 (64.5)
		Master’s degree	67 (13.4)
		PhD degree	43 (8.6)
Employment status		Student	47 (9.4)
		Employed	237 (49.2)
		Unemployed	30 (6.0)
		Own business	8 (1.6)
		Retired	82 (16.4)
		House wife	77 (15.4)
Family members		single	12 (2.4)
		2	36 (7.4)
		3–4	110 (22.0)
		> 4	327 (67.9)
COVID-19 pandemic related parameters			
Change in family income	Yes		147 (30.5)
	No		334 (69.4)
Weight change since quarantine started	Yes, increased		202(41.9)
	Yes, decreased		178 (37.0)
	No change, weight is stable		101 (20.9)
Change in dietary habits	Quantity of meal increased		171 (35.6)
	Quantity of meal decreased		102 (21.2)
	Dependent on home cooking for main meals		277 (57.6)
	Dependent on outside foods for main meals (e.g., takeaway from restaurants)		13 (2.7)
	Frying is the main cooking method		117 (24.3)
	Boiling, broiling, and grilling are the main cooking methods		163 (33.9)
Change in lifestyle habits	Water consumption has increased		184 (38.3)
	Water consumption has decreased		91 (18.9)
	Tea and coffee consumption have increased		128 (26.6)
	Tea and coffee consumption have decreased		46 (9.6)
	Physical activity has increased		84 (17.4)
	Physical activity has decreased		209 (43.4)
	Sleeping hours have increased		147 (30.5)
	Sleeping hours have decreased		122 (25.3)
	Time spent on screens has increased		292 (60.7)
	Time spent on screens has decreased		28 (5.8)
	Number of cigarettes smoked has increased		29 (6.0)
	Number of cigarettes smoked has decreased		22 (4.5)

### Calculation of sample size

Calculating the size of the study sample was done using the Epi Info online calculator to identify the total study subjects required to reach the chosen confidence level [16]. Based on the Saudi Arabian General Authority for Statistics in 2019 [17] and earlier research done in Saudi Arabia [18], 468 participants were minimally required to achieve the study objectives, given that the estimated dropout rate was 20%, with a confidence level of 99%, a margin error of 5%, and a design effect of 1.

### Statistical analysis

Reporting categorical parameters was as frequencies (N) and percentages (%) and reporting continuous parameters was as mean ± standard deviation (SD). Parameters were compared between baseline (since quarantine started) and at the end of the study. Determining the differences in parameters within weight loss and weight gain groups was done using paired sample T-test (normal continuous parameters) and Wilcoxon-signed rank test (non-normal continuous parameters). Determining the differences in parameters between weight gain and weight loss groups was done using independent sample T-test (normal continuous parameters) and Mann–Whitney U-test (non-normal continuous parameters). To determine the effect of different categorical parameters of interest on weight change, Chi-Square test was applied. To identify potential risk factors for weight change, multinomial logistic regression analysis was used for different categorical parameters of interest as independent predictors to weight change, with either weight loss or gain as the dependent parameter and stable weight as a reference category. Determining significance level was done if P value was less than 0.05. Statistical analysis was undertaken with the help of IBM SPSS statistical software, Version 27 (Armonk, NY, USA).

## Results

### General characteristics

A total of 502 respondents completed this cross-sectional study, however, only 481 were included in the analysis (mean age of the respondents is 41.9 ± 14.2, of which 184 (38.2%) were male, and 297 (61.7%) were females), while 21 (4.1%) were excluded for either being living outside KSA, or for being younger than the inclusive age group (i.e., < 18 years old). Table 2 displays the characteristics of the studied population.

### Weight change related to Covid-19 pandemic and Ramadan fasting

**Fig. 1:**
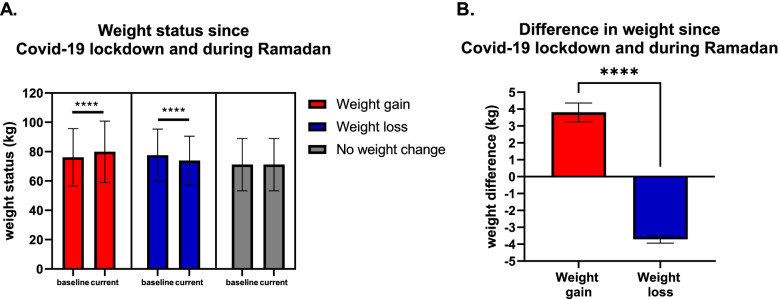
Weight status among participants during Ramadan and Covid-19 lockdown. Data represent frequency (*n* = 481) of (  ) people who gain weight, (  ) people who lost weight, and (  ) people who did not have any weight change. Paired t-test was used **A** to compare weight change within groups of before Ramadan and Covid-19 lockdown and after this period. Unpaired t-test was used **B** to compare weight differences between groups. Data at which values differed significantly, *p < 0.05, **p < 0.01, ***p < 0.001

Since the quarantine started and during Ramadan, 42.4% of the participants had gained weight (mean weight 79.9 ± 20.8 kg (was76.1 ± 19.5 kg at baseline), *p* < 0.0001), 38.4% of the participants had lost weight (mean weight 73.9 ± 16.6 kg (was77.6 ± 17.7 kg at baseline), *p* < 0.0001), and 23.4% had no weight change (Fig. 1). When comparing the weight differences between groups, a statistical significance difference was found between people who lost weight and those who gained weight (mean weight lost 3.7 ± 3.0 kg, mean weight gained 3.8 ± 7.8 kg, *p* < 0.0001) (Fig. 1).

### Dietary habits change related to Covid-19 pandemic

Of the participants who lost weight and the participants who gained weight, 64.6% and 52.9% have considered home cooking during lockdown and Ramadan, respectively. None of the participants who lost weight and only 4.5% of participants who gained weight have considered food from outside home (i.e., takeaways). When analysing the relationship between home-cooking and weight status, it was found that home cooking has significantly associated with weight loss (*P* = 0.01), but not with weight gain (*P* = 0.96).

Of participants who lost weight and the participants who gained weight, 44.4% and 36.1% have considered not to change the quantity of their meals (i.e., not to increase nor decrease) during lockdown and Ramadan, respectively. Moreover, 21.9% of the participants who lost weight and 51% of participants who gained weight have increased their quantity of food consumption either during their regular meals or by adding extra meals. When analysing the relationship between quantity of food consumed and weight status, it was found that not changing the quantity of meals consumed has significantly associated with weight loss (*P* = 0.006). On the other hand, increasing quantity of food consumption either during regular meals or by adding extra meals has associated significantly with weight gain (*P* < 0.001).

**Fig. 2:**
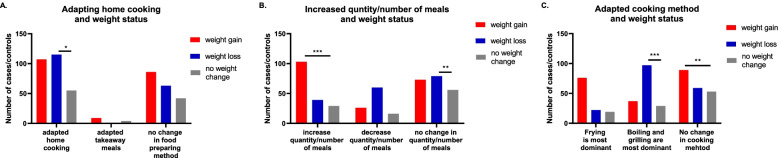
Relationship between dietary habits and weight status among participants During Ramadan and Covid-19 lockdown. Data represent frequency (*n* = 481) of (  ) people who gain weight, (  ) people who lost weight, and (  ) people who did not have any weight change. Chi square test was used **A** to assess the relationship between relying on home cooked meal and weight change between groups, **B** to assess the relationship between increased quantity of meals and weight change between groups, and **C** to assess the relationship between adapting different cooking techniques and weight change between groups. Data at which values differed significantly, *p < 0.05, **p < 0.01, ***p < 0.001

Of participants who lost weight and the participants who gained weight, 54.5% and 18.3% have considered dominating boiling and grilling as the main cooking methods during lockdown and Ramadan, respectively. Moreover, 33.1% of the participants who lost weight and 44.1% of participants who gained weight have considered not to change their cooking method during lockdown and Ramadan. When analysing the relationship between dominated cooking method and weight status, it was found that dominating boiling and grilling as the main cooking methods has associated significantly with weight loss (*P* < 0.001). On the contrary, not changing the cooking method has associated significantly with weight gain (*P* = 0.003) (Table 3, Fig. 2).

**Table 3:** Changes in dietary habits related to Covid-19 pandemic in their correspondence weight group (*n* = 481)

	weight gain (*n* = 202), n(%)	weight loss (*n* = 178), n(%)	no weight change (*n* = 101), n(%)
**Dependent on home cooking versus takeaway meals**			
Dependent on home cooking	107 (52.97)	115 (64.60)	55 (54.45)
Dependent on takeaway meals	9 (4.45)	0	4 (3.96)
No change in food preparation	86 (42.57)	63 (35.39)	42 (41.58)
**Increase versus decrease quantity/number of meals**			
increase quantity/number of meals	103 (50.99)	39 (21.91)	29 (28.71)
decrease quantity/number of meals	26 (12.87)	60 (33.70)	16 (15.84)
no change in quantity/number of meals	73 (36.13)	79 (44.38)	56 (55.44)
**Frying versus boiling and grilling as dominant cooking methods**			
Frying is most dominant	76 (37.62)	22 (12.35)	19 (18.81)
Boiling and grilling are most dominant	37 (18.31)	97 (54.49)	29 (28.71)
No change in cooking method	89 (44.05)	59 (33.14)	53 (52.47)

### Lifestyle habits change related to Covid-19 pandemic

Of participants who lost weight and the participants who gained weight, 46.6% and 35.1% have considered increasing their water intake during lockdown and Ramadan, respectively. When analysing the relationship between water intake and weight status, it was found that increasing water intake has significantly associated with weight loss (*P* = 0.006). On the other hand, the amount of water intake did not associate with weight gain (*P* = 0.54).

Of participants who lost weight and the participants who gained weight, 63.5% and 59.9% have not changed their amount of caffeine intake during lockdown and Ramadan than before these periods, respectively. When analysing the relationship between caffeine intake and weight status, no significant association was found with weight loss (*P* = 0.29) neither with weight gain (*P* = 0.08).

Of participants who lost weight and the participants who gained weight, 37.1% and 34.2% have not changed their physical activity level during lockdown and Ramadan, respectively. Moreover, 33.1% of participants who lost weight and 56.4% of participants who gained weight have decreased their level of physical activity during lockdown and Ramadan. When analysing the link between physical activity level and weight status, it was found that continuing on the same physical activity level as before lockdown and Ramadan has associated significantly with weight loss (*P* = 0.002). On the contrary, decreasing the level of physical activity has associated significantly with weight gain (*P* = 0.003).

Of participants who lost weight and the participants who gained weight, 41.0% and 40.6% have not changed their sleep habits during lockdown and Ramadan than before these periods, respectively. When analysing the relationship between sleep habits and weight status, no significant association was found with weight loss (*P* = 0.46) neither with weight gain (*P* = 0.33).

Of participants who lost weight and the participants who gained weight, 56.2% and 65.3% have increased their screen time/habit during lockdown and Ramadan than before these periods, respectively. When analysing the relationship between screen time/habit and weight status, no significant association was found with weight loss (*P* = 0.15) neither with weight gain (*P* = 0.38).

**Fig. 3:**
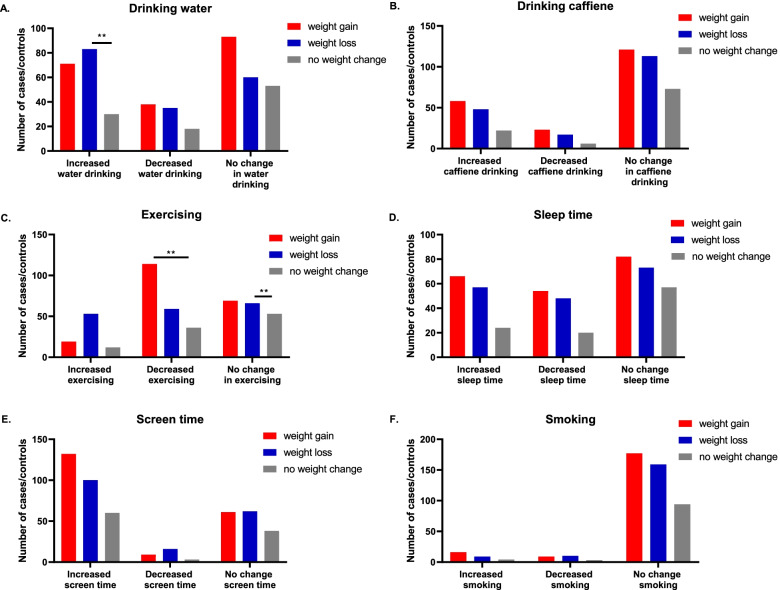
Relationship between lifestyle habits and weight status among participants During Ramadan and Covid-19 lockdown. Data represent frequency (*n* = 481) of (  ) people who gain weight, (  ) people who lost weight, and (  ) people who did not have any weight change. Chi square test was used **A** to assess the relationship between amount of water drinking and weight change between groups, **B** to assess the relationship between increased quantity of meals and weight change between groups, **C** to assess the relationship between amount of exercising and weight change between groups, **D** to assess the relationship between sleep time and weight change between groups, **E** to assess the relationship between screen duration and weight change between groups, **F** to assess the relationship between smoking and weight change between groups. Data at which values differed significantly, *p < 0.05, **p < 0.01, ***p < 0.001

Of participants who lost weight and the participants who gained weight, 89.3% and 87.6% have not changed their smoking habits during lockdown and Ramadan than before these periods, respectively. When analysing the relationship between smoking habits and weight status, no significant association was found with weight loss (*P* = 0.53) neither with weight gain (*P* = 0.33) (Table 4, Fig. 3).

**Table 4:** Changes in lifestyle habits related to Covid-19 pandemic in their correspondence weight group (*n* = 481)

	**weight gain (*n* = 202), n(%)**	**weight loss (*n* = 178), n(%)**	**no weight change (*n* = 101), n(%)**
**Increased versus decreased water drinking**			
Increased water drinking	71 (35.14)	83 (46.62)	30 (29.70)
Decreased water drinking	38 (18.8)	35 (19.66)	18 (17.82)
No change in water drinking	93 (46.03)	60 (33.70)	53 (52.47)
**Increased versus decreased caffeine drinking**			
Increased caffeine drinking	58 (28.71)	48 (26.96)	22 (21.78)
Decreased caffeine drinking	23 (11.38)	17 (9.55)	6 (5.94)
No change in caffeine drinking	121 (59.90)	113 (63.48)	73 (72.27)
**Increased versus decreased level of physical activity**			
Increased level of physical activity	19 (9.40)	53 (29.77)	12 (11.88)
Decreased level of physical activity	114 (56.43)	59 (33.14)	36 (35.47)
No change in level of physical activity	69 (34.15)	66 (37.07)	53 (52.47)
**Increased versus decreased sleep time**			
Increased sleep time	66 (32.67)	57 (32.02)	24 (23.76)
Decreased sleep time	54 (26.73)	48(26.96)	20 (19.80)
No change sleep time	82 (40.59)	73 (41.01)	57 (56.40)
**Increased versus decreased screen time**			
Increased screen time	132 (65.34)	100 (56.17)	60 (59.40)
Decreased screen time	9 (4.45)	16 (8.98)	3 (2.97)
No change screen time	61 (30.19)	62 (34.83)	38 (37.62)
**Increased versus decreased smoking**			
Increased smoking	16 (7.92)	9 (5.61)	4 (3.96)
Decreased smoking	9 (4.45)	10 (5.61)	3 (2.97)
No change smoking	177 (87.62)	159 (89.32)	94 (93.06)

### Multivariate regression analysis to identify the most probable risk factor for weight change

In order to identify the most probable risk factors that directly influence weight status, multivariate regression analysis was done. The analysis showed that physical activity is one factor that can protect against weight gain (OR = 1.03 with *P* = 0.008), while increasing the quantity of meals and not adapting healthy cooking methods can both be considered as contributing factors to weight gain (OR = 1.03 with *P* = 0.009, and OR = 1.03 with *P* = 0.004, respectively).

## Discussion

To the best of the author’s knowledge, this research is the first to highlight the impact of both COVID-19 quarantine and Ramadan fasting on weight status, and the association between weight change and lifestyle factors including dietary habits, physical activity, water intake, screen and sleep times, and smoking, and the underlying factors that attributed to the change in weight status among the population living in the KSA. In general, this study has found that increasing number of meals and not adapting healthy cooking methods as the most dominant cooking technique of food consumption are the main risk factors for weight gain, while exercising as the main protective factor.

### Weight change related to Covid-19 pandemic and Ramadan fasting

Around 42% of the recruited participants have had around 5% lockdown and Ramadan weight gain in the current findings, which is clinically significant amount of weight to be gained with its substantial predisposition of comorbidities over subsequent years [19]. Thus, protection from unfortunate health effects will likely happen by managing weight gain during COVID-19 [20, 21]. This result is in line with other researches during COVID-19 quarantine internationally [3, 6, 11, 22–25], and nationally [18]. Fewer people with weight gain have been reported in other studies in comparison to the current study [26].

On the other hand, around 38% of the recruited participants have had around 5% lockdown and Ramadan weight loss in the current findings, which is clinically significant amount of weight to be lost, as it is associated with physiological and biochemical benefits [27]. This finding is in line with the result in the Chinese study [25] and other local ones [18, 26]. Other papers have reported fewer people with weight loss [5, 6, 22, 28]. When comparing between the groups, the percentage of participants who gained weight was minimally, but significantly, higher than the percentage of participants who lost weight.

Although the outcomes of the present research are in line with previous research concerning weight change during quarantine, they do not coincide entirely with previous research concerning weight change during Ramadan. It was shown that intermittent fasting (as in Ramadan) can cause significant weight loss in many previous studies, including a 2020 systematic review and meta-analysis [29–31]. However, these studies have been conducted earlier to the times of COVID-19 crisis, where people were less stressed and more living their normal life without being confined to their homes. High level of stress and low level of life satisfaction can both influence weight gain [32, 33]. Traditionally, specific ritual and social behaviours are known to take place during Ramadan; including social gatherings during main meals in a buffet style and prayers and worship. During the pandemic, many families could not have applied many of the traditions they used to, given the governmental lockdown and home confinement policies and the fear of contracting infection. This have massively affected people’s life satisfaction and anxiety levels, which can subsequently be manifested in weight gain [34]. Obesity can be considered as a condition of chronic low-grade inflammation, given the immunomodulatory effects of the adipokine secreted from the adipose tissue [35], which can downregulate both the immune and adaptive immune responses, increasing the body’s vulnerability to infections and decrease its responsiveness to antimicrobial and antiviral drugs, and vaccinations [36]. Moreover, the reduction of protective cardiorespiratory reserves due to excess ectopic fat can have detrimental effects on lung function [37, 38]. As a consequence, individuals with obesity are threatened from developing serious illnesses including COVID-19 infection, if infected, or other more severe forms of respiratory failure. Thus, global public health and community nutrition campaigns targeted towards sustaining normal weights, especially during times of quarantine, are required.

### Dietary habits change related to Covid-19 pandemic

Analysis has showed that both increasing the quantity of meals and not adapting healthy cooking methods as the dominant technique of food preparation are directly associated factors with weight gain. The significance levels extend to the same factors when considering multivariate regression analysis, which indicates the importance of these factors to be considered as risk factors for weight gain during home confinement and Ramadan.

On the contrary, home cooking, not increasing the quantity of meals consumed, and adapting healthy cooking methods as the dominant technique of food preparation (as boiling and grilling) are all directly associated factors with weight loss. However, these factors did not reach significance levels when considering multivariate regression analysis, indicating the partial influence of these factors on weight loss during lockdown and Ramadan.

It can be concluded from the current results that home confinement is a serious dietary threat, especially to individuals with obesity and overweight, as more problematic eating behaviors are exhibited in these groups, including frequent food consumption and overeating in the absence of hunger [39, 40]. Such behaviors might get further stimulated during lockdown owing to, often, unlimited availability to large amounts of foods during the extended stay at home, as previous research showed [41]. This can lead to a disturbance in the time-restricted feeding window; a known factor that has a positive effect in dysmetabolism and obesity and promote robust metabolic cycles [42]. Previous research further supports the link between higher amounts of food consumption with the global lockdown [2, 3, 28, 37].

Foods cooked at home are considered healthier and/or lower in calories than foods away from home (as restaurants) [43]. However, 53% of participants of the current study who gained weight have adapted home-cooking for their food intake than before the lockdown. Nevertheless, 51% and 44% of participants of the same weight group have reported an increase in quantity of foods and/or meals consumed and not acquiring healthy cooking methods as boiling and grilling, respectively. These findings could be explained by the fact that some cooking methods may not be considered as healthy, especially if people added large amount of fat and sugar which would add extra amounts of calories, and would increase food palatability and thus making it more appealing in such challenging circumstances. In addition, such foods are traditionally known to be consumed in Ramadan, even before the pandemic, and are known to increase the risk of infections [44–46]. Generally, these results are comparable to those in Bakhsh et al. study, and are further explained by studies in Spain and Italy, where they found that homemade cakes and breads were the most common Google search terms and were higher consumed than before lockdown [3, 4, 18, 28]. Thus, a similar trend in cooking and food choices may have been found in Saudi Arabia. Nevertheless, 64.6% of participants who lost weight have relied on consuming home-cooked meals, which seems to be healthier and less caloric way of cooking, as 54.5% of participants from the same group have considered healthy cooking methods (as boiling and grilling) as the main technique of food preparation. This outcome is in line with the Spanish study, where they found better adherence to healthy cooking methods for food preparation during the lockdown [4].

It is proposed that a healthy diet is considered as a crucial factor of the individual risk assessment and management strategy during pandemics as COVID-19, given its significant effect against responding to an infectious agent [47]. This protective effect arises from the immunomodulatory effects of several phyto-, micro-, and macronutrients that have profound roles in immunocompetence. In contrast, nutritional deficiencies have been known to increase host vulnerability to infections and other severe diseases [48, 49]. For instance, during outbreaks as COVID-19, the Mediterranean diet was proposed to be followed due to its role in boosting the immune system [50], which was feasible and well-adhered to by Italian and Spanish population [4, 28]. Moreover, following healthy well-balanced diet during Ramadan is recommended, given its immunomodulatory effects against infections and to reduce or maintain weight [51]. On the contrary, it has been suggested that unhealthy diets negatively affect host susceptibility to infections and subsequent recovery [52, 53]. Subsequently, a vicious cycle of weight gain and increased risk of infection will cause both COVID-19 and to obesity be regarded as two colliding public health pandemics [54, 55]. Therefore, it is mandatory for people with overweight or obesity to stick to an individual risk management strategy that includes healthy well-balanced diet [47, 56].

### Lifestyle habits change related to Covid-19 pandemic

Analysis has showed that decreasing physical activity level is directly associated with weight gain. However, this factor does not reach significance level when considering multivariate regression analysis, indicating the partial effect of physical activity level on weight gain during lockdown and Ramadan.

On the other hand, increasing water intake, and maintaining physical activity level as before lockdown and Ramadan periods are all directly associated with weight loss. The significance level extends only to the physical activity levels when considering multivariate regression analysis, which indicates the importance of this factor to be considered as a protective element against weight gain during lockdown and Ramadan.

This finding support recent research, as it is well established the dose–response relationship between weight loss and physical activity levels [57]. Moreover, the findings of the present research are in line with earlier research that show the dramatical global influence of COVID-19 quarantine on lifestyle activities, including the physical activity involvement [58, 59]. This is logical to expect, given the diverse governmental confinement policy on movement restrictions during COVID-19 pandemic which will directly affect the participation in physical activity [60]. It was shown previously in China that differences in physical activity level was associated with both different regional policies on confinement and socio-economic levels [61]. The decrease in physical activity level is even more affected due to Ramadan fasting, where it is reported previously to be reduced during the holy month [62]. Thus, addressing these factors when designing physical activity interventions via the involvement of remote dietitian services, social media campaigns, and health care authorities are essential for such pandemic in the future.

It is interesting that the present study found no association between screen time and weight change. However, it is stated in a previous review that any link between sedentary lifestyle, including screen time, and weight gain may not appear as a causal relationship [63]. The current study findings coincide with the mentioned review, where screen time may not be a predictor for weight change during lockdown and Ramadan.

Noteworthy, the current study took place in Saudi Arabia and was conducted in a relatively short time, as suggested previously [64]. However, sharing a part of the middle east on how the lockdown resulted from the epidemic during Ramadan can influence the dietary and lifestyle behaviors and weight, as in Saudi Arabia, for the first time can provide valuable insights to the neighboring gulf and Arabian countries. Nevertheless, the current outcomes should be considered for future-similar circumstances aimed towards the prevention and preparation if any lockdown-incidents are to be necessitated. Future consideration should include the permissibility to not to fast and the fluctuation in weight as a result of water retention and hormonal changes during the menstrual cycle in child-bearing aged women, where excluding these days and this change in weight are needed before including this group in the analysis. Moreover, self-reported dietary and lifestyle behaviors and weight recall at the same time point based on an online and anonymous questionnaire rather than standardized baseline measures to objectively confirm the data prior to and following the study timeline, in addition to the mixed genders and the broad age groups can all be underlined as study limitations. Thus, the outcomes of the current research should be considered as a rough measure rather than an accurate value. Overcoming the mentioned weaknesses were, however, impossible baring in mind the challenges of running such a study in a restricted period of time as Ramadan in a national lockdown.

## Conclusion

To the best of the author’s knowledge, this research is the first to highlight the impact of weight change and the related dietary and lifestyle habits during Ramadan of COVID-19 quarantine in Saudi Arabia. Number of people who gained weight were significantly higher than weight loss. The weight gain was shown to be associated with decreased level of physical activity while increased quantity of meals and not adapting healthy cooking method can both be considered as potential risk factors. The results also showed an association between weight loss and adapting home cooking and healthy cooking methods, increasing water drinking, and not increasing quantity of meals consumed while not changing the level of physical activity in everyday life can be considered as protective factors. Assessing these changes during Ramadan of COVID-19 quarantine provided valuable perspective on the health and wellbeing of Saudi Arabia citizens. These findings should be considered in future studies to question the persistence of Covid-19 related weight and habit changes.

## Supplementary Information

**Additional file 1.** English version of the questionnaire.

## Data Availability

All data generated or analysed during this study are included in this published article.

## References

[1] Wang C, Pan R, Wan X, Tan Y, Xu L, McIntyre RS (2020). A longitudinal study on the mental health of general population during the COVID-19 epidemic in China. Brain Behav Immun.

[2] Ammar A, Brach M, Trabelsi K, Chtourou H, Boukhris O, Masmoudi L (2020). Effects of COVID-19 home confinement on eating behaviour and physical activity : results of the. Nutrients.

[3] Scarmozzino F, Visioli F (2020). Covid-19 and the subsequent lockdown modified dietary habits of almost half the population in an Italian sample. Foods.

[4] Rodríguez-Pérez C, Molina-Montes E, Verardo V, Artacho R, García-Villanova B, Guerra-Hernández EJ (2020). Changes in dietary behaviours during the COVID-19 outbreak confinement in the Spanish COVIDiet study. Nutrients.

[5] Drywień ME, Hamulka J, Zielinska-Pukos MA, Jeruszka-Bielak M, Górnicka M (2020). The COVID-19 pandemic lockdowns and changes in body weight among Polish women. A cross-sectional online survey PLifeCOVID-19 Study. Sustainability.

[6] Sidor A, Rzymski P (2020). Dietary choices and habits during COVID-19 lockdown: experience from Poland. Nutrients.

[7] Popkin BM, Du S, Green WD, Beck MA, Algaith T, Herbst CH (2020). Individuals with obesity and COVID-19: a global perspective on the epidemiology and biological relationships. Obes Rev.

[8] Ministry of Interior. A royal order to limit the spread of the Corona virus from seven in the evening until six in the morning for a period of 21 days from the evening of Monday 28 Rajab March 23. Ministry of Interior. 2020. Available from: https://www.moi.gov.sa/wps/portal. Cited 2021 Sep 14.

[9] Faris MAIE, Alsibai J, Jahrami HA, Obaideen AA, Jahrami HA, Obaideen AA (2020). Impact of Ramadan diurnal intermittent fasting on the metabolic syndrome components in healthy, non-athletic Muslim people aged over 15 years: a systematic review and meta-analysis. Br J Nutr.

[10] Harder-Lauridsen NM, Rosenberg A, Benatti FB, Damm JA, Thomsen C, Mortensen EL (2017). Ramadan model of intermittent fasting for 28 d had no major effect on body composition, glucose metabolism, or cognitive functions in healthy lean men. Nutrition.

[11] Zachary Z, Forbes B, Lopez B, Pedersen G, Welty J, Deyo A, et al. Self-quarantine and weight gain related risk factors during the Covid-19 pandemic. Obes Res Clin Pract. 2020;14(3):210-216. 10.1016/j.orcp.2020.05.004 PMC7241331 32460966

[12] Pellegrini M, Ponzo V, Rosato R, Scumaci E, Goitre I, Benso A (2020). Changes in weight and nutritional habits in adults with obesity during the “lockdown” period caused by the COVID-19 virus emergency. Nutrients.

[13] Khan MMA, Nor NM, Mamat NM, Mohd-Shukri NA, Abu Bakar WAM (2016). Fasting in Islam: a combination of spiritual elevation and prevention of diseases. Int Med J Malays.

[14] Dong E, Du H, Gardner L (2020). An interactive web-based dashboard to track COVID-19 in real time. Lancet Infect Dis.

[15] Alfawaz H, Amer OE, Aljumah AA, Aldisi DA, Enani MA, Aljohani NJ (2021). Effects of home quarantine during COVID-19 lockdown on physical activity and dietary habits of adults in Saudi Arabia. Sci Rep.

[16] Division of Health Informatics and Surveillance (DHIS) (2018). Centers for disease control and prevention.

[17] Saudi General Authority for Statistics. Population by age groups ,and gender. Saudi General Authority for Statistics Mid 2020. 2020;35013414. Available from: https://www.stats.gov.sa/sites/default/files/Population%20by%20Age%20Groups%20%2Cand%20Gender_0.pdf.

[18] Bakhsh MA, Khawandanah J, Naaman RK, Alashmali S (2021). The impact of COVID-19 quarantine on dietary habits and physical activity in Saudi Arabia: a cross-sectional study. BMC Public Health.

[19] Dutton GR, Kim Y, Jacobs DR, Li X, Loria CM, Reis JP, et al. 25-year weight gain in a racially balanced sample of U.S. adults: The CARDIA study. Obesity. 2016;24(9):1962–8. Available from: http://onlinelibrary.wiley.com/journal/10.1002/(ISSN)1930-739X%0A. http://ovidsp.ovid.com/ovidweb.cgi?T=JS&PAGE=reference&D=emed18&NEWS=N&AN=611908692 10.1002/oby.21573 PMC5004783 27569121

[20] Palaiodimos L, Kokkinidis DG, Li W, Karamanis D, Ognibene J. Since January 2020 Elsevier has created a COVID-19 resource centre with free information in English and Mandarin on the novel coronavirus COVID- 19 . The COVID-19 resource centre is hosted on Elsevier Connect , the company ’ s public news and information. 2020.

[21] World Health Organization. Obesity and overweight. World Health Organization. 2020. Available from: https://www.who.int/news-room/fact-sheets/detail/obesity-and-overweight. Cited 2021 Sep 14.

[22] Fernandez-Rio J, Cecchini JA, Mendez-Gimenez A, Carriedo A (2020). Weight changes during the COVID-19 home confinement. Effects on psychosocial variables. Obes Res Clin Pract.

[23] Reyes-Olavarría D, Latorre-Román PÁ, Guzmán-Guzmán IP, Jerez-Mayorga D, Caamaño-Navarrete F, Delgado-Floody P (2020). Positive and negative changes in food habits, physical activity patterns, and weight status during covid-19 confinement: associated factors in the chilean population. Int J Environ Res Public Health.

[24] He M, Xian Y, Lv X, He J, Ren Y (2021). Changes in body weight, physical activity, and lifestyle during the semi-lockdown period after the outbreak of COVID-19 in China: an online survey. Disaster Med Public Health Prep.

[25] Xia QS, Wu F, Zhao Y, Huang ZY, Dong H, Xu LJ, et al. Risk factors related to weight gain for chines during home connement in COVID-19 pandemic: an observational retrospective study. 2020;1–18. 10.21203/rs.3.rs-55697/v1

[26] Al-Musharaf S, Aljuraiban G, Bogis R, Alnafisah R, Aldhwayan M, Tahrani A (2021). Lifestyle changes associated with COVID-19 quarantine among young Saudi women: a prospective study. PLoS One.

[27] Wing RR, Lang W, Wadden TA, Safford M, Knowler WC, Bertoni AG (2011). Benefits of modest weight loss in improving cardiovascular risk factors in overweight and obese individuals with type 2 diabetes. Diabetes Care.

[28] di Renzo L, Gualtieri P, Pivari F, Soldati L, Attinà A, Cinelli G (2020). Eating habits and lifestyle changes during COVID-19 lockdown: an Italian survey. J Transl Med.

[29] Khaled BM, Belbraouet S (2009). Effect of Ramadan fasting on anthropometric parameters and food consumption in 276 type 2 diabetic obese women. Int J Diabetes Dev Ctries.

[30] Daradkeh G, Abuzaid H, Al-muhannadi A, Abuhmaira M, Khalili A, Acido H (2021). Effect of Ramadan fasting on body composition and dietary intake : a prospective study in the State of Qatar. J Nutr Food Sci.

[31] Jahrami HA, Alsibai J, Clark CCT, Faris MAIE (2020). A systematic review, meta-analysis, and meta-regression of the impact of diurnal intermittent fasting during Ramadan on body weight in healthy subjects aged 16 years and above. Eur J Nutr.

[32] Geiker NRW, Astrup A, Hjorth MF, Sjödin A, Pijls L, Markus CR (2018). Does stress influence sleep patterns, food intake, weight gain, abdominal obesity and weight loss interventions and vice versa?. Obes Rev.

[33] Korkeila M, Kaprio J, Rissanen A, Koskenvuo M, Sörensen TIA (1998). Predictors of major weight gain in adult Finns: stress, life satisfaction and personality traits. Int J Obes.

[34] Lima CKT, de Carvalho PMM, de Lima IAAS, de Nunes JVAO, Saraiva JS, de Souza RI (2020). The emotional impact of Coronavirus 2019-nCoV (new Coronavirus disease). Psychiatry Res.

[35] de Lorenzo A, Gratteri S, Gualtieri P, Cammarano A, Bertucci P, di Renzo L (2019). Why primary obesity is a disease?. J Transl Med.

[36] Dhurandhar NV, Bailey D, Thomas D (2015). Interaction of obesity and infections. Obes Rev.

[37] Sattar N, McInnes IB, McMurray JJV (2020). Obesity is a risk factor for severe COVID-19 infection: multiple potential mechanisms. Circulation.

[38] Malavazos AE, Corsi Romanelli MM, Bandera F, Iacobellis G (2020). Targeting the adipose tissue in COVID-19. Obesity.

[39] Opichka K, Smith C, Levine AS (2019). Problematic eating behaviors are more prevalent in African American Women who are overweight or obese than African American women who are lean or normal weight. Family Community Health.

[40] Yau YHC, Potenza MN (2013). Stress and eating behaviors. Minerva Endocrinol.

[41] Rolls BJ, Roe LS, Meengs JS (2007). The effect of large portion sizes on energy intake is sustained for 11 days. Obesity.

[42] Zarrinpar A, Chaix A, Panda S (2016). Daily eating patterns and their impact on health and disease circadian rhythms and metabolism. Trend Endocrinol Metab.

[43] Todd JE, Mancino L, Lin B hwan, Jessica E. The impact of food away from home on adult diet quality Visit Our Website To Learn More ! Cataloging Record : United States Journal of Agriculture. 2010;(90).

[44] Myles IA (2014). Fast food fever: reviewing the impacts of the Western diet on immunity: discovery service for Endeavour College of Natural Health Library. Nutr J.

[45] Rafie C. Fasting During Ramadan : Nutrition and Health Impacts and Food Safety Recommendations. Virginia Tech [Internet]. 2016;10. Available from: https://www.pubs.ext.vt.edu/content/dam/pubs_ext_vt_edu/HNFE/HNFE-351/HNFE-351-PDF.pdf.

[46] Frost G, Pirani S (1987). Meal frequency and nutritional intake during Ramadan: a pilot study. Hum Nutr Appl Nutr.

[47] Gasmi A, Noor S, Tippairote T, Dadar M, Menzel A, Bjørklund G (2020). Individual risk management strategy and potential therapeutic options for the COVID-19 pandemic. Clin Immunol.

[48] Chandra RK (1996). Nutrition, immunity and infection: From basic knowledge of dietary manipulation of immune responses to practical application of ameliorating suffering and improving survival. Proc Natl Acad Sci U S A.

[49] Bhaskaram P. Micronutrient malnutrition, infection, and immunity: an overview. Nutrition Reviews. 2002;60(suppl 5):S40–S45. 10.1301/00296640260130722. 10.1301/00296640260130722 12035857

[50] Muscogiuri G, Barrea L, Savastano S, Colao A (2020). Nutritional recommendations for CoVID-19 quarantine. Eur J Clin Nutr.

[51] British Nutrition Foundation (2019). Healthy Ramadan 2021.

[52] Naja F, Hamadeh R (2020). Nutrition amid the COVID-19 pandemic: a multi-level framework for action. Eur J Clin Nutr.

[53] Butler MJ, Barrientos RM (2020). Since January 2020 Elsevier has created a COVID-19 resource centre with free information in English and Mandarin on the novel coronavirus COVID- 19. The COVID-19 resource centre is hosted on Elsevier Connect, the company ’ s public news and information. Brain Behav Immun.

[54] Ryan DH, Ravussin E, Heymsfield S (2020). COVID 19 and the patient with obesity – the editors speak out. Obesity.

[55] Clemmensen C, Petersen MB, Sørensen TIA (2020). Will the COVID-19 pandemic worsen the obesity epidemic?. Nat Rev Endocrinol.

[56] Kalligeros M, Shehadeh F, Mylona EK, Benitez G, Beckwith CG, Chan PA (2020). Association of obesity with disease severity among patients with coronavirus disease 2019. Obesity.

[57] Slentz CA, Duscha BD, Johnson JL, Ketchum K, Aiken LB, Samsa GP (2004). Effects of the amount of exercise on body weight, body composition, and measures of central obesity: STRRIDE - a randomized controlled study. Arch Intern Med.

[58] de Oliveira NL, Elsangedy HM, Tavares VDDO, Teixeira CVLS, Behm DG, da Silva-Grigoletto ME (2020). #TrainingInHome - Home-based training during COVID-19 (SARS-COV2) pandemic: physical exercise and behavior-based approach. Revista Brasileira de Fisiologia do Exercício.

[59] Ammar A, Chtourou H, Boukhris O, Trabelsi K, Masmoudi L, Brach M (2020). Covid-19 home confinement negatively impacts social participation and life satisfaction: a worldwide multicenter study. Int J Environ Res Public Health.

[60] Kriaucioniene V, Bagdonaviciene L, Rodríguez-Pérez C, Petkeviciene J (2020). Associations between changes in health behaviours and body weight during the covid-19 quarantine in lithuania: the lithuanian covidiet study. Nutrients.

[61] Hossain MM, Sultana A, Purohit N (2020). Mental health outcomes of quarantine and isolation for infection prevention: a systematic umbrella review of the global evidence. Epidemiol Health.

[62] Alghamdi AS, Alghamdi KA, Jenkins RO, Alghamdi MN, Haris PI (2020). Impact of Ramadan on physical activity and sleeping patterns in individuals with type 2 diabetes: the first study using fitbit device. Diabetes Therapy.

[63] Biddle SJH, Bengoechea García E, Pedisic Z, Bennie J, Vergeer I, Wiesner G (2017). Screen time, other sedentary behaviours, and obesity risk in adults: a review of reviews. Current Obesity Reports..

[64] Geldsetzer P (2020). Use of rapid online surveys to assess people’s perceptions during infectious disease outbreaks: a cross-sectional survey on COVID-19. J Med Internet Res..

