# Supplementary material for: The impact of Ramadan during COVID-19 confinement on weight, dietary, and lifestyle habits in the Kingdom of Saudi Arabia: a cross-sectional study
Source: BMC Public Health. 2022 Aug 30;22:1649. doi: 10.1186/s12889-022-13953-9 (PMC9427176; doi:10.1186/s12889-022-13953-9)
Supplement: Supplementary file 1 — Additional file 1. English version of the questionnaire. [file 12889_2022_13953_MOESM1_ESM.docx]

**The impact of Ramadan during COVID-19 confinement on weight, dietary, and lifestyle habits in Saudi Arabia: a cross-sectional study**

**Supplementary Information**

Additional file 1 of the English version of the questionnaire used to investigate the impact of Ramadan during COVID-19 confinement on weight, dietary, and lifestyle habits in Saudi Arabia: a cross-sectional study

**Additional file 1: English version of the questionnaire.**

1. Place of living

- Inside Saudi Arabia

- Outside Saudi Arabia

1. Sex

- Male

- Female

1. Age

- 18-25 years old

- 26-35 years old

- 36-45 years old

- > 45 years old

1. Hight in cm?
2. Current weight in kg?
3. Usual weight before Covid-19 confinement in kg?
4. Education

- High school

- Diploma

- Bachelor degree

- Master degree

- PhD degree

1. Work status during the Corona pandemic

- Student

- Employed

- Unemployed

- Own business

- Retired

- House wife

1. Family members

- single

- 2 members

- 3-4 members

- >4 members

1. Was there a change in family income as a result of Covid-19 pandemic?

- Yes

- No

1. Was there a change in weight since quarantine started?

- Yes, increased

- Yes, decreased

- No change, weight is stable

1. Was there a change in dietary habits since quarantine started?
   1. With regards to: quantity of meal

- Quantity of meal increased

- Quantity of meal decreased

- No change

- 1. With regards to: source of main meal

- Dependent on home cooking for main meals

- Dependent on outside foods for main meals (e.g., takeaway from restaurants)

- No change

- 1. With regards to: main cooking method

- Frying is the main cooking method

- Boiling, broiling, and grilling are the main cooking methods

- No change

1. Was there a change in lifestyle habits since quarantine started?
   1. With regards to: water consumption

- Water consumption has increased

- Water consumption has decreased

- No change

- 1. With regards to: tea and coffee consumption

- Tea and coffee consumption have increased

- Tea and coffee consumption have decreased

- No change

- 1. With regards to: physical activity

- Physical activity has increased

- Physical activity has decreased

- No change

- 1. With regards to: sleeping hours consumption

- Sleeping hours have increased

- Sleeping hours have decreased

- No change

- 1. With regards to: time spent on screens

- Time spent on screens has increased

- Time spent on screens has decreased

- No change

- 1. With regards to: smoking consumption

- Number of cigarettes smoked has increased

- Number of cigarettes smoked has decreased

- No change
